# A Complex Interplay: Navigating the Crossroads of Tobacco Use, Cardiovascular Disease, and the COVID-19 Pandemic: A WHF Policy Brief

**DOI:** 10.5334/gh.1334

**Published:** 2024-07-01

**Authors:** Regina Dalmau, Abdullah M. Alanazi, Monika Arora, Amitava Banerjee, Eduardo Bianco, Diann E. Gaalema, Fastone M. Goma, Koji Hasegawa, Maki Komiyama, Mónica Pérez Ríos, Jeffrey Willett, Yunshu Wang

**Affiliations:** 1 University Hospital La Paz, Madrid, Spain; 2 King Saud Bin Abdulaziz University for Health Sciences, Saudi Arabia; 3 King Abdullah International Medical Research Center, Saudi Arabia; 4 Public Health Foundation of India, India; 5 University College London, United Kingdom; 6 Frank Foundation for International Health, Uruguay; 7 University of Texas Medical Branch, United States; 8 Centre for Primary Care Research, Zambia; 9 National Hospital Organization Kyoto Medical Center, Japan; 10 University of Santiago de Compostela, Spain; 11 Health Research Institute of Santiago de Compostela, Spain; 12 American Heart Association, United States; 13 World Heart Federation, Switzerland

**Keywords:** Tobacco, cardiovascular disease, COVID-19

## Abstract

The Coronavirus Disease 2019, commonly referred to as COVID-19, is responsible for one of the deadliest pandemics in human history. The direct, indirect and lasting repercussions of the COVID-19 pandemic on individuals and public health, as well as health systems can still be observed, even today.

In the midst of the initial chaos, the role of tobacco as a prognostic factor for unfavourable COVID-19 outcomes was largely neglected. As of 2023, numerous studies have confirmed that use of tobacco, a leading risk factor for cardiovascular and other diseases, is strongly associated with increased risks of severe COVID-19 complications (e.g., hospitalisation, ICU admission, need for mechanical ventilation, long COVID, etc.) and deaths from COVID-19. In addition, evidence suggests that COVID-19 directly affects multiple organs beyond the respiratory system, disproportionately impacting individuals with comorbidities. Notably, people living with cardiovascular disease are more prone to experiencing worse outcomes, as COVID-19 often inherently manifests as thrombotic cardiovascular complications. As such, the triad of tobacco, COVID-19 and cardiovascular disease constitutes a dangerous cocktail.

The lockdowns and social distancing measures imposed by governments have also had adverse effects on our lifestyles (e.g., shifts in diets, physical activity, tobacco consumption patterns, etc.) and mental well-being, all of which affect cardiovascular health. In particular, vulnerable populations are especially susceptible to tobacco use, cardiovascular disease and the psychological fallout from the pandemic. Therefore, national pandemic responses need to consider health equity as well as the social determinants of health.

The pandemic has also had catastrophic impacts on many health systems, bringing some to the brink of collapse. As a result, many health services, such as services for cardiovascular disease or tobacco cessation, were severely disrupted due to fears of transmission and redirection of resources for COVID-19 care. Unfortunately, the return to pre-pandemic levels of cardiovascular disease care activity has stagnated. Nevertheless, digital solutions, such as telemedicine and apps, have flourished, and may help reduce the gaps.

Advancing tobacco control was especially challenging due to interference from the tobacco industry. The industry exploited lingering uncertainties to propagate misleading information on tobacco and COVID-19 in order to promote its products. Regrettably, the links between tobacco use and risk of SARS-CoV-2 infection remain inconclusive. However, a robust body of evidence has, since then, demonstrated that tobacco use is associated with more severe COVID-19 illness and complications. Additionally, the tobacco industry also repeatedly attempted to forge partnerships with governments under the guise of corporate social responsibility. The implementation of the WHO Framework Convention on Tobacco Control could address many of the aforementioned challenges and alleviate the burden of tobacco, COVID-19, and cardiovascular disease. In particular, the implementation of Article 5.3 could protect public health policies from the vested interests of the industry.

The world can learn from the COVID-19 pandemic to better prepare for future health emergencies of international concern. In light of the impact of tobacco on the COVID-19 pandemic, it is imperative that tobacco control remains a central component in pandemic preparedness and response plans.

## Introduction

The COVID-19 pandemic has had, and will continue to have, an immense impact on our daily lives. The public health and social measures imposed by governments worldwide, including social distancing and lockdowns, are bound to leave lasting repercussions on our societies. Undeniably, the health, social and economic burdens of the pandemic have made us feel more vulnerable than ever.

Tobacco, widely recognised as a major risk factor for cardiovascular morbidity and mortality, contributes to approximately 17% of all cardiovascular deaths globally [1]. Exposure to tobacco not only accelerates the progression of atherosclerosis and increases the risk of acute thrombotic events, but also impairs the immune system, weakening its ability to combat infectious agents [2]. Unfortunately, due to the severity of the first pandemic waves and the near collapse of many healthcare systems, the role of tobacco as a prognostic factor was frequently overlooked. In fact, tobacco use was massively underreported in medical records during the early stages of the pandemic [3]. Amidst the chaos, the tobacco industry capitalised on lingering uncertainties to disseminate unsubstantiated and misleading information about purported protective effects of tobacco-smoking against SARS-CoV-2 infections, thereby undermining national responses to the pandemic [4]. Nonetheless, the deadly interplays between tobacco and COVID-19 must not be underestimated. Multiple independent studies have confirmed that tobacco smoking is associated with more severe cases of COVID-19, leading to a higher incidence of acute respiratory failure as well as an increased need for mechanical ventilation [5].

A growing body of evidence has demonstrated that both tobacco and COVID-19 critically impact the outcomes of various health conditions and overall quality of life [2,5]. A myriad of studies have established that people living with cardiovascular and respiratory conditions and/or associated risk factors tend to develop more severe COVID-19-related illness and experience worse outcomes [6]. Furthermore, emerging data also suggests that SARS-CoV-2 infection significantly increases the risk of thrombotic events [7]. As such, the combination of tobacco, COVID-19 and cardiovascular disease constitutes a dangerous cocktail.

This position paper aims to review the intricate interactions between tobacco, COVID-19, and cardiovascular disease from both health and societal perspectives. It intends to outline the direct, indirect and long-term effects of COVID-19 with a specific emphasis on the role of cardiovascular disease and its associated risk factors, notably tobacco, within a learning health system framework [8]. Lastly, the policy brief also seeks to provide guidance on tobacco regulation and control in the post-pandemic era.

## Tobacco, COVID-19, and Cardiovascular Disease

The novel coronavirus disease 2019, commonly referred to as COVID-19, is caused by the severe acute respiratory syndrome coronavirus 2 (SARS-CoV-2). Countless studies have shown that the virus adversely affects multiple organs beyond the respiratory system [9]. In particular, it is well-known that the angiotensin-converting enzyme 2 (ACE2)—the receptor for SARS-CoV-2—is expressed not only in the mucosal epithelial cells and alveolar tissue of the airways, but also widely in the heart and cardiovascular system [10]. In fact, COVID-19 mainly manifests as thrombotic cardiovascular complications, which may be further exacerbated by tobacco use, in addition to respiratory failure resulting from pneumonia [11].

Risk factors that increase the likelihood of severe illness and death from COVID-19 include smoking, advanced age, cardiovascular disease, hypertension, diabetes, chronic respiratory disease and malignancy [12]. Experts have suggested that smoking stimulates the activation of ACE2, which in turn facilitates the entry of the virus into the cells as well as its spread within the body [13]. Myocardial injury, as evidenced by elevated blood troponin levels, has also been shown to be strongly associated with poor prognosis in COVID-19 patients [7]. Possible mechanisms of myocardial injury in COVID-19 include ischaemia stemming from circulatory and respiratory failure, small coronary artery thrombotic occlusion in the epicardium or myocardium due to increased coagulation capacity, and systemic inflammation or myocarditis caused by direct binding of the virus to ACE2, which is abundantly expressed in the heart [14]. Sustained immune activation during the viral infection is also hypothesised to increase the risk of developing dilated cardiomyopathy in COVID-19 patients [15]. The acute inflammatory response triggered by the infection can deteriorate cardiac function and worsen symptoms in heart failure patients [16]. Furthermore, it is known that some cases of myocarditis have recurrent relapses and remissions. Therefore, patients at high risk for myocardial damage should be monitored in the long-term after SARS-CoV-2 infection to track potential progression to heart failure [17].

In addition, factors such as smoking, advanced age and cardiovascular disease are also associated with intravascular dysfunction and increased coagulability, which may be contributing mechanisms to adverse outcomes in COVID-19. As such, treatment of COVID-19-associated hypercoagulability abnormalities (i.e., in coagulation and fibrinolysis) are closely related to the aetiology of COVID-19 and have a significant impact on prognosis. Therefore, it is recommended to measure D-dimer, fibrinogen, prothrombin time and platelet count in all COVID-19 patients [11].

**Figure 1:**
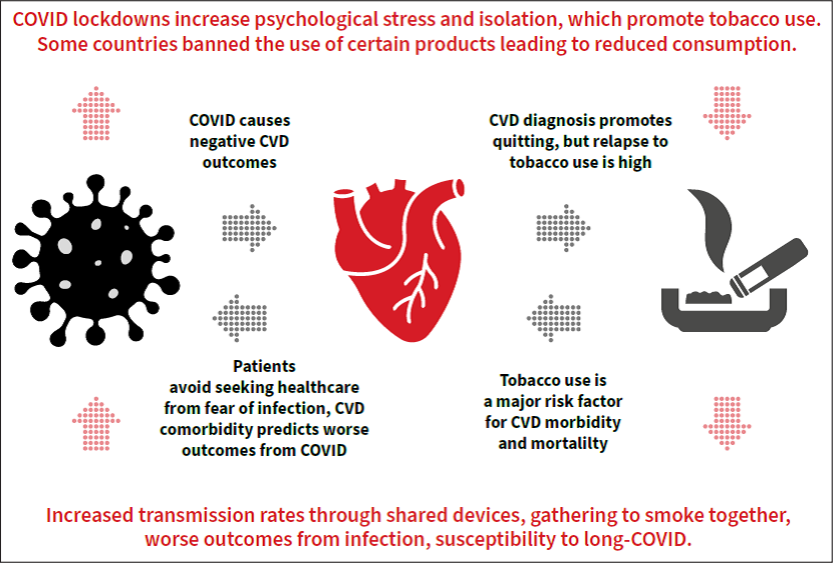
Direct and indirect interplays between tobacco use, cardiovascular disease, and COVID-19.

Furthermore, the measures implemented in response to the epidemic, although necessary, have had drastic consequences on our lifestyles (Figure 1). For instance, restrictions or prohibitions on outings often led to significant reductions in physical activity, increased propensities for unhealthy diets, limited access to health promotion services (e.g., tobacco cessation services, etc.), mental health deterioration, as well as increased use of tobacco and alcohol, all of which are closely associated with cardiovascular diseases, especially coronary artery disease and heart failure [18,19,20].

## Impact of the COVID-19 Pandemic on Tobacco Consumption

### Pandemic response and mental health

The COVID-19 pandemic compelled countries to enforce strict sanitation regimes and social distancing measures, including lockdowns. These interventions, alongside fears surrounding SARS-CoV-2 infection, generated large amounts of stress in the population. As a result, the mental health of society as a whole has been severely affected by the global epidemic, to the extent that the term “COVID depression” was coined [21]. In fact, the pandemic has led to a substantial increase in the number of people showing signs of depression and cases of suicides worldwide [22].

According to a survey conducted by the United Nations, the percentage of people who felt distress during the COVID-19 pandemic rose to 35% in China, 45% in the United States, and 60% in Iran [23]. In addition, a Centers for Disease Control and Prevention online survey of 5,412 US adults, conducted in June 2020, found that 40.9% of respondents reported experiencing some form of mental disorder, such as symptoms of anxiety or depressive disorders. Of these respondents, 10.7% indicated that they had seriously contemplated suicide in the past 30 days [24]. In Japan, suicide rates increased for 4 consecutive months from July 2020. In particular, October 2020 reported a staggering 39.9% increase from the previous year, with an alarming 82.6% increase among women, in particular [25]. In general, prolonged curfews following major natural disasters are known to have significant negative impacts on people’s psyche, as exemplified by the surge in incidence of mental illness following lockdown measures [26]. The above findings suggest that individuals may be prone to poor and/or irrational decision-making under high-stress circumstances, such as a declared state of emergency [27].

Mental health issues often coincide with the initiation, continuation, and escalation in use of addictive substances (e.g., tobacco, alcohol, drugs, etc.) as well as with negative changes in diet and physical activity; this raises concerns about potential harmful downstream effects, such as an increase in noncommunicable diseases and specifically, cardiovascular disease [28].

### Pandemic response and tobacco consumption

The COVID-19 pandemic has had a noticeable influence on smoking behaviours. Factors such as stress and poor mental health are known to increase smoking frequency, quantity and dependence, as well as trigger relapses in ex-smokers [29]. While some countries have reported an increase in cigarette consumption, others have observed varied usage patterns at the population level [30].

A study in the United Kingdom involving current smokers (n = 329) reported that 25.2% smoked more cigarettes than usual, 50.9% smoked the same amount and 20.2% smoked less. The study also found significantly worse mental health, anxiety, stress and depressed mood among those who smoked more, suggesting a pandemic-induced deterioration in mental health and psychosocial well-being, as well as an associated increase in smoking [31]. In a sample of individuals in the US from vulnerable populations (i.e., those with lower-socioeconomic status or with psychiatric conditions, N = 332) current smokers reported smoking more cigarettes during the pandemic compared to the pre-pandemic period, even leaving their homes specifically to purchase cigarettes [32]. In Japan, among current smokers (n = 1,000), 18% smoked more cigarettes than usual, 69.6% smoked the same amount and 11.4% smoked less [33]. Elevated stress due to changes in daily life as well as the social environment was reported as the primary cause of the shift in consumption pattern.

A number of studies have reported no significant change in the total number of cigarettes smoked, as increases in some demographics may have been offset by decreases in others. In fact, it is plausible that disadvantaged populations (e.g., those with mental health disorders, etc.) either continued to smoke or smoked more, while more advantaged groups seized the opportunity to quit tobacco. Those who smoked less were often motivated by a desire to mitigate the risk of severe COVID-19 illness and to protect family members from second-hand smoke [34,35,36].

## Impact of the COVID-19 Pandemic on the Burden of Tobacco-Related Diseases

### Access to health services during the COVID-19 pandemic: delayed diagnosis and treatment of acute and chronic NCD conditions

During the COVID-19 pandemic, and particularly during the first waves, access to healthcare facilities was severely disrupted. Tobacco use is a risk factor for many of the acute and chronic conditions that are exacerbated by poor access to the healthcare system, with special implications for vulnerable populations (e.g., homeless, migrants, etc). According to the *WHO Pulse Survey on the Continuity of Essential Health Services During the COVID-19 Pandemic*, disruptions of essential health services were reported by 90% of countries. These included services for communicable diseases, noncommunicable diseases (with a 69% reduction in diagnosis and treatment), mental health and nutrition, as well as reproductive, maternal, newborn, child and adolescent health. Significant reductions in outpatient care attendance were reported by 76% of countries and potentially life-saving emergency services were disrupted in almost a quarter of countries. These disruptions were caused by a combination of demand- and supply-side factors, with 76% of the countries reporting a lower demand in outpatient care attendance due to lockdowns (48%) or financial difficulties (33%). Cancellation of elective services was the most commonly reported issue on the supply side (66%), in addition to staff redistribution to provide COVID-19 care, lack of sufficient personal protective equipment for healthcare professionals, closures of services or healthcare facilities, and interruption of supplies for health products [37].

During the first waves, most hospitals were overwhelmed by acute COVID-19 admissions, and primary care nearly collapsed in some countries. Many healthcare professionals were infected or diverted from their usual activities to provide COVID-19 related care. As a result, the scarcity in human resources had become a major barrier to dealing with the health crisis. For instance, preventive activities, such as smoking cessation programmes, were interrupted for several months. Many surgical services, notably cancer surgeries, were also cancelled or delayed. In addition, the follow-ups of many chronic conditions were interrupted, which led to severe consequences in terms of health outcomes [38].

During the lockdowns, a significant decline in hospitalisations for cardiovascular events was recorded in many countries. Different studies have reported a drop in the number of patients admitted for acute coronary syndromes (ACS) as well as a reduction in the number of cardiac catheterisations [39,40,41,42]. A retrospective analysis of consecutive patients admitted for ACS performed in 15 hospitals from northern Italy with primary percutaneous coronary intervention programmes during the first months of the pandemic, showed a decline in ACS admissions compared to the same period the previous year (i.e., 13.3 admissions per day versus 18.0, incidence rate ratio, 0.74; 95% confidence interval [CI], 0.66 to 0.82; P < 0.001). In addition, a further reduction in ACS admissions was reported after the national lockdown [40]. Even though the exact reasons for such a decrease are difficult to determine, it has been reported that some patients avoided seeking medical attention during the first months of the pandemic due to fear of contagion. Social distancing and avoidance of medical care are potential behavioural explanations, as has been reported in previous pandemics [43]. In a multicentre retrospective observational registry conducted in 75 specific ACS care centres in Spain, comparing patients treated before and after COVID-19, the number of suspected ST-elevation myocardial infarction (STEMI) patients treated decreased by 27.6% after the COVID-19 outbreak, as the number of confirmed STEMI fell by 22.7%. In addition, there were no changes in reperfusion strategy or time from first medical contact to reperfusion, despite patients presenting longer ischaemic times (233 vs 200 minutes, p < 0.001). The belated consultations of health services were presumably the main cause of delay to reperfusion. It is worth noting that early management of acute STEMI is crucial in reducing cardiovascular morbidity and mortality. In fact, in the Spanish study, in-hospital mortality was significantly higher during COVID-19 (7.5% vs 5.1%; unadjusted OR, 1.50; 95%CI, 1.07–2.11; P <.001) [42]. Evidence from the United Kingdom suggests that return to pre-pandemic levels of cardiovascular disease care activity for numerous procedures has been slow [39].

Secondary prevention was also severely impacted. Access to cardiac rehabilitation and structured secondary prevention programmes was compromised, undermining the recent progress in delivering secondary prevention. In a cross-sectional study with a survey completed by 1,062 cardiac rehabilitation programmes across 70 countries, 75% reported ceasing temporarily their activity or interrupting the admission of new patients [44]. Additionally, many cardiac rehabilitation centres, especially those that served rural and socially vulnerable populations, closed permanently due to interruptions in payments for preventive services [45]. Smoking cessation is an important goal in secondary prevention and a core component of cardiac rehabilitation; thus, cessation efforts were further stymied by these closures. The interruption of smoking cessation programmes, coupled with the stressful social and economic conditions of the pandemic, have been a barrier to advancing tobacco cessation efforts.

### Interruption of smoking cessation services

The COVID-19 pandemic emphasised the importance of preserving respiratory as well as cardiovascular health, providing a unique window of opportunity to further promote tobacco cessation and control. Unfortunately, excessive pressure on healthcare systems resulted in drastic interruptions to smoking cessation services.

A cross-sectional US study analysing the cessation behaviour of adults aged 18 years and above reported that, for the first time in a decade, the annual past-year quit attempt prevalence decreased (i.e., from 65.2% in 2019 to 63.2% in 2020), with the largest relative decreases being among individuals aged 45 to 64 years, individuals with 2 or more comorbidities, Black individuals, individuals with lower educational attainment, and women. Concurrently, retail sales of nicotine replacement therapies across 31 US states decreased by an average of 1% to 13% compared to expected sales. Possible explanations for this decrease include healthcare disruptions and pandemic-induced stress [46].

In response to the challenges imposed by the pandemic, many healthcare systems have established or strengthened digital health solutions to facilitate access to health professionals and counterbalance limited access to healthcare facilities. In particular, telemedicine has been especially well-integrated into our post-COVID practices. Telehealth can play a crucial role in providing advice and monitoring smoking cessation attempts. It can also facilitate access to smoking cessation interventions for socially disadvantaged populations or individuals living in remote areas. The promotion of tobacco cessation through apps and digital public health campaigns can play a key role in reducing the burden of tobacco in the post-COVID-19 era. According to recent studies, many smokers expressed interest in accessing various forms of cessation assistance during the pandemic [47].

Indeed, the COVID-19 pandemic presumably also provided smokers with additional incentives and motivation to quit tobacco. In fact, a recent study reported a positive effect of the pandemic on smoking cessation rates in patients attending a specialised clinic in Turkey, with successful cessation rates raising from 23% to 31% during the pandemic [48].

Health authorities and regulators worldwide need to provide a synergistic response to the intertwined public health issues of COVID-19 and the tobacco epidemic. Effective smoking cessation programmes, telemedicine tools, and smoking cessation campaigns will play a key role in the post-COVID era.

### The case for sustaining NCD and tobacco cessation services through health emergencies

COVID-19 is one the most devastating pandemics in human history, disproportionately affecting those with co-morbid diseases [5,49]. Data suggest that smokers infected with COVID-19 are at greater risks of longer hospital stays, ICU admission and need for mechanical ventilation [5,49,50,51]. In fact, several studies have corroborated that patients with COVID-19 who are former or current smokers face significantly higher mortality rates in comparison to never smokers [5,49,50,51,52,53,54,55,56]. In addition, smoking was also identified as a risk factor for increased mortality among COVID-19 patients with underlying chronic health conditions, such as obesity, cancer, chronic obstructive pulmonary disease and cardiovascular disease [5,49,57,58,59]. Some studies also illustrated that COVID-19-associated mortality among smokers may vary based on sex, with female smokers exhibiting higher mortality rates than male smokers [56].

Although some reports suggested that smoking was not significantly associated with mortality among patients with COVID-19 [60], various meta-analysis collectively demonstrated that mortality rates are statistically higher among current and/or former smokers affected by COVID-19 [5,57]. It should be evident that smoking remains a risk factor for increased disease severity, morbidity and mortality. As such, comprehensive preventive and treatment services should be fully utilised to lessen the burden of tobacco and COVID-19, especially among vulnerable patients [5,57].

## Smoking As a Prognostic Factor of COVID-19

### Biological evidence and plausibility

Biologically plausible pathways suggest that tobacco use could potentially increase susceptibility to, and severity of, COVID-19. Longstanding evidence indicates that smokers, especially current ones, are more susceptible to viral and bacterial respiratory infections, both in mild and more severe forms [61]. Cigarette smoking aggravates respiratory infections, leading to an increased risk of susceptibility to, and severity of, pathogens such as influenza and tuberculosis [62,63]. The mechanisms connecting cigarette smoking and respiratory illnesses include both immune suppression and physiological changes, such as abnormal cilia function [64]. In addition, many of the harmful chemicals present in tobacco products, along with those generated during aerosolisation through heating or combustion, have deleterious effects on the respiratory system, thereby potentially increasing both susceptibility to and severity of SARS-CoV-2 [65]. Conversely, evidence suggests that the immune system starts to recover and reduces vulnerability to infections within just one month of quitting tobacco [66,67].

### Epidemiologic evidence from prior coronavirus outbreaks

Epidemiological studies of prior coronavirus outbreaks, specifically SARS-1 and MERS, found no evidence to support the hypothesis that smoking offers protection against coronaviruses. In fact, the prevailing body of research suggests that smoking contributes to both a greater risk of illness onset and increased severity. Despite rumours that emerged in 2003 suggesting that smokers were less likely to contract SARS-CoV-1, subsequent studies demonstrated no difference between smokers and nonsmokers in their risks of developing the illness [68]. In addition, studies of another coronavirus strain, the Middle East Respiratory Syndrome (MERS-CoV), revealed that smoking significantly increased the probability of contracting the illness [69]. Additionally, it was found that the case fatality rate among smokers was twice that of non-smokers [70].

### Limitations of clinical records and case series designs

Research exploring the relationships between tobacco use and SARS-CoV-2 have primarily relied on clinical data from hospitals and other healthcare settings. Even before the COVID-19 pandemic, multiple studies highlighted significant concerns and gaps regarding accurate documentation of tobacco use in clinical records. For instance, a Finnish study found that only 60% of patients admitted to hospital between 2010 and 2016 had tobacco use documented in their clinical records. Furthermore, the likelihood of tobacco use being documented varied based on the patients’ illness and disease diagnoses [3].

The COVID-19 pandemic, which overwhelmed many health systems, likely made the collection of accurate clinical data on tobacco use even more challenging for clinicians and other health personnel. For example, a review found that 80% (186 out of 233) of studies published between February and August 2020 had ‘poor’ documentation, with more than 20% of clinical records lacking information on tobacco use status [71]. These incomplete or inaccurate clinical records pose substantial methodological issues for studying the relationships between tobacco use and COVID-19.

Another methodological concern is the predominant use of, and reliance on, ‘case series’ medical research designs in the majority of published studies on smoking and COVID-19. These studies draw from a pool of subjects engaged in clinical care, without the potential for control or comparison groups. While much of the research regarding smoking and COVID-19 relies on individuals who are actively engaged in clinical settings, researchers have pointed out that smokers participating in these clinical studies may not be representative of the general population. For instance, Simons and colleagues suggest that uninfected smokers may be overrepresented in SARS-CoV-2 testing data because they are more likely to present with COVID symptoms (e.g., coughing, altered sense of smell or taste, etc.) that meet community criteria for testing [71].

The potential underreporting of current smoking is evident across many studies using clinical data. For example, out of 74,439 case report forms of patients with COVID-19 submitted to the US Centers for Disease Control and Prevention during the early phase of the pandemic, only 1.3% of patients were classified as current smokers, far lower than what would be expected from the general population [72]. Studies like this do not provide a clear understanding of the association between smoking and COVID-19. Without study designs that better ensure accurate documentation of tobacco use in clinical records and better represent the general population, it will be challenging to determine the extent to which smoking is a prognostic factor for SARS-CoV-2.

### Is smoking protective against SARS-CoV-2 infection?

Despite substantial methodological issues, as outlined above, several studies published in the early stages of the pandemic suggested that smoking decreased the risk of infection from SARSCoV-2 [73]. Pro-tobacco constituents amplified these findings, with the media seemingly eager to highlight these potentially counter-intuitive preliminary findings [74].

A rigorous review of the studies that were published from the start of the pandemic through August 2021 found inconsistent and contradictory results with regard to the relationship between smoking and SARS-CoV-2 infection [65]. The review identified 26 studies suggesting that cigarette smoking was associated with reduced SARS-CoV-2 infection rates. However, it also identified 20 studies associating smoking with increased SARS-CoV-2 infection rates, as well as 7 studies that found no association between smoking and SARS-CoV-2 infection. Despite presenting inconsistent results pertaining to infection, the review provided clear evidence that smokers were more likely to experience more severe disease outcomes.

While additional research is required to fully understand the exact relationship between smoking and infection from SARS-CoV-2, it has been well-documented that smoking is a major risk factor for the incidence of respiratory infection, including other coronaviruses such as MERS, as well as for the severity of disease progression [75,76].

### Tobacco and long COVID

Most patients afflicted by COVID-19 will experience symptoms relief within 2–6 weeks. However, in some cases, after recovery from initial infection, various symptoms may persist for 2–3 months, or even longer, as sequelae. These lingering symptoms, often referred to as long COVID, can occur irrespective of age and severity of COVID-19.

Symptoms of long COVID can be diverse, including fatigue, arthralgia, muscle pain, cough, phlegm, shortness of breath, chest pain, hair loss, memory impairment, poor concentration, insomnia, headache, depression, olfactory disturbance, dysgeusia (i.e., taste disorder), palpitations, diarrhoea, abdominal pain, sleep disturbance, and muscle weakness. Although precise data are not yet available, as diagnostic criteria and methods of investigation vary from country to country, it is estimated that 10–20% of patients experience long COVID [77]. Approximately two-thirds of these sequelae will disappear within three months, but in some cases, recovery may extend to years. Concerningly, long COVID has been associated with significant psychiatric issues, including suicidal ideation and behaviour [78].

Various theories have been posited regarding the pathogenic mechanisms of long COVID, including direct damage to virus-infected tissues (especially the lungs), progressive and continued inflammation due to immune dysregulation, vascular damage and tissue ischaemia caused by increased blood coagulation and subsequent thrombus formation, dysregulation of the renin-angiotensin system caused by viral infection, and post-intensive care syndrome in critically ill patients [79].

Notably, smoking and e-cigarettes, apart from being independent risk factors for more severe clinical manifestations and worsening of COVID-19, also contribute to the development of long COVID. These correlations have been reported in several large cohort studies [80,81,82]. The risk of sequelae in smokers is reported to be 8.39 times higher than in individuals who never smoked [83]. Furthermore, smoking has also been identified as an independent risk factor for delayed recovery from long COVID [84]. Therefore, implementing tobacco control measures is critical in preventing and managing long COVID effectively.

### Tobacco use and risk of severe COVID-19

Risk factors for severe COVID-19 outcomes include smoking, advanced age and pre-existing conditions, such as cardiovascular disease, diabetes, malignant neoplasms and chronic respiratory disease. A multivariate study from China identified four key factors contributing to COVID-19 severity: a history of smoking, temperature upon admission, respiratory failure and age 60 years or older [85]. Of these, the odds ratio associated with smoking history was the largest at 14 (confidence interval [1.6–45]; p = 0.018), surpassing the odds ratios for the other aggravating factors (8.5–9.0). Reports on 1,099 infected individuals in China indicate that 12.4% of current smokers and 23.8% of former smokers experienced severe outcomes, including admission to intensive care units, ventilator use, or death. In contrast, only 4.7% of nonsmokers experienced similar outcomes [75]. Furthermore, the proportion of patients with severe outcomes was 21.2% for current smokers and 42.9% for past smokers, both higher than the 14.5% for nonsmokers [75]. The increased severity of COVID-19 symptoms in former smokers could be explained by the fact that they may have been, on average, older than current smokers. Another possibility would be that past smokers may have more comorbidities and, consequently, an increased risk of serious illness compared to current smokers (e.g., quitting tobacco after contracting a disease, etc).

As noted above, smoking is an important determinant of COVID-19 severity. It is important to highlight that most underlying diseases associated with severe COVID-19 outcomes, such as cardiovascular disease, chronic obstructive pulmonary disease and diabetes mellitus, are all strongly associated with tobacco use.

## Tobacco Control During the COVID-19 Crisis

The COVID-19 pandemic has uncovered a need for greater global emphasis on public health infrastructures, including a stronger focus on tobacco control measures. Countries with more fragile health systems encountered significant challenges in managing the various COVID-19 outbreaks within their jurisdictions. In many instances, the redeployment of limited public health resources for COVID-19 management led to the disruption or suspension of other essential health services, such as tobacco prevention and cessation interventions.

The COVID-19 crisis led some countries to enforce tobacco bans as a mean to curb the spread of the virus and ease the strain on healthcare systems [86,87]. Botswana, India and South Africa instituted complete, albeit temporary, tobacco sales bans as part of their lockdown measures [86,88,89]. Other countries, such as Italy, Pakistan, Saudi Arabia, Spain and the United Arab Emirates, imposed partial bans on tobacco products to address the escalating healthcare burden [86]. These bans were mostly enacted temporarily under essential goods restrictions [88,90,91]. However, some countries, including Kenya, classified tobacco products as essential goods [92].

### Tobacco control opportunities and missed opportunities

The pandemic has created a unique set of challenges and opportunities for tobacco control efforts. It has spotlighted the importance of preserving respiratory and cardiovascular health, highlighting the urgency of promoting smoking cessation and minimising tobacco use.

As restrictions eased, some countries implemented measures to protect their populations against exposure to second-hand smoke. For instance, in some cases, policies were implemented to prohibit smoking in outdoor areas of bars and restaurants, with customers required to smoke at a designated distance. Unfortunately, these protective measures were often withdrawn as the de-escalation of restrictions progressed.

The pandemic has also disrupted ongoing smoking cessation programs, leading to missed opportunities to further alleviate both the burden of COVID-19 and tobacco-related diseases. With healthcare systems overwhelmed by the influx of COVID-19 cases, many healthcare professionals were diverted from their usual responsibilities to manage the pandemic, leaving other health services, such as smoking cessation interventions, neglected [38]. However, these obstacles also spurred the development of innovative strategies, including digital solutions such as telemedicine, public health campaigns, and smoking cessation apps, to provide support for individuals trying to quit smoking [46]. Telemedicine and smoking cessation apps can play key roles in providing critical advice and support to individuals trying to quit, especially for those in remote areas or vulnerable populations.

### Tobacco control and lessons learnt from the COVID-19 pandemic

The correlation between tobacco use and increased severity of, and mortality from, COVID-19 among hospitalised patients highlights an urgent need for countries to accelerate the implementation of the WHO Framework Convention on Tobacco Control (FCTC) [93]. In fact, the implementation of the WHO FCTC would reduce fatalities from tobacco, COVID-19 and potential future pandemics as many articles within the treaty are highly relevant in the context of the COVID-19 pandemic. For instance, Article 5.3 addresses interference from the tobacco industry, Article 8 protects nonsmokers from second-hand exposure, Article 6 outlines taxation measures which could serve to raise income for national pandemic responses, and Article 14 promotes tobacco cessation [93,94,95].

Many countries have demonstrated both the capacity and political will to enact key tobacco control measure throughout the pandemic. For instance, Spain temporarily expanded its smoke-free policies to include outdoor areas [93]. This political resolve was formalised with the quasi-unanimous adoption of a *Declaration on WHO FCTC and Recovery from the COVID-19 Pandemi*c at the Ninth Session of the Conference of the Parties to the WHO FCTC in 2021 [4]. Nevertheless, it is essential that countries maintain and extend these measures beyond the COVID-19 crisis [96].

Furthermore, the pandemic has also exacerbated the health equity and equality issues associated with tobacco use [94,97]. Studies indicate that the social determinants of health, particularly education, significantly impact both tobacco consumption as well as cardiovascular disease morbidity and mortality. In fact, disadvantaged groups, such as populations with lower socio-economic status and health literacy, are more susceptible to developing cardiovascular disease [98,99] and more prone to using tobacco products [100,101]. In the context of the COVID-19 pandemic, families under financial strain and household with lower levels of education experienced worse living conditions (e.g., poor housing, increased exposure to tobacco smoke, etc.) and displayed detrimental health-related behaviour (e.g., physical inactivity, unhealthy diets, etc.) during lockdowns [97]. Therefore, pandemic preparedness and response plans must consider health equity and the social determinants of health.

### Case study of India

In India, tobacco-related deaths account for 9.5% of all deaths at the country level, with 48% of these deaths caused by cardiovascular disease, most commonly due to ischaemic heart disease [102]. Furthermore, tobacco use is responsible for the development of cardiovascular disease among younger people, accounting for 26% of CVD cases in the age group of 30–44 years [102]. Studies indicate that smokeless tobacco (ST) use is also a risk factor for cardiovascular disease [103]. According to the INTERHEART study estimates, over 6 million disability-adjusted life years (DALYs) and 258,006 lives lost due to ischaemic heart disease can be attributed to ST use in 2017 [103].

#### Prohibition on spitting

During the COVID-19 pandemic, countries tailored their national responses and tobacco control policies according to their specific contexts. India has a unique tobacco use situation as the primary form of consumption is smokeless, with 21% of adults being ST users and 11% using combusted forms of tobacco [104]. ST use is often associated with spitting behaviour, which raised alarm due to the higher risk of transmission linked to spitting [105]. As a response, the use of ST in public was prohibited under the National Directive for COVID-19 Management, with fines implemented to discourage spitting, thereby reducing the risk of COVID-19 transmission.

#### Quitting tobacco during COVID-19

A study conducted in two metropolitan cities (i.e., Delhi and Chennai) in India assessed the tobacco cessation behaviour among 801 adult tobacco users, with a history of any form of tobacco use, following a prohibition on the sales and use of tobacco products during the first lockdown in March 2020. The study revealed that 11% of tobacco users managed to quit during the lockdown, with a median of two quit attempts per users. Individuals with higher levels of knowledge about the harmful effects of tobacco use and COVID-19 were significantly more likely to quit tobacco use (OR 2.2) and reported more quit attempts compared to those with poor knowledge on the issue. It was also reported that users who had access to tobacco products were less likely to quit use compared to those who had no access. The study concluded that the access restriction measures introduced by the Government of India, coupled with accurate knowledge about the harmful effects of tobacco use and COVID-19, created a conducive environment to quit tobacco [106].

#### Reduced frequency of use

During the pandemic, the government implemented a ban on the sales of cigarettes and other tobacco products, while conducting a campaign to raise awareness of the potential connections between tobacco use and severe COVID-19 outcomes. Findings from this study conducted in Delhi and Chennai revealed significant changes in the frequency of smoking (i.e., bidis and cigarettes) and smokeless tobacco use. The data showed a significant decrease in all forms of tobacco use during the lockdown compared to the pre-lockdown period. This reduction may be primarily attributed to reduced access to tobacco products and increased awareness [107].

## Tobacco Industry Interference

The tobacco industry continues to be the primary obstacle to the progression of tobacco control globally [108]. Unfortunately, the advent of the COVID-19 pandemic has done nothing to curtail its tireless efforts to obstruct life-saving policies. Conversely, the industry initiated several misinformation campaigns, throughout the pandemic, to influence public perceptions about the link between tobacco use and COVID-19 [86].

In June 2020, the World Health Organization issued a statement linking tobacco smoking with more severe COVID-19 outcomes, urging smokers to quit to mitigate their risk of severe complications [86]. The tobacco industry immediately reacted by launching multiple misinformation campaigns, mainly via social media, to sway public opinion on the issue [86]. One such campaign propagated the false idea that nicotine had protective effects against COVID-19 [86]. As previously noted, the confusion surrounding the association between tobacco use, nicotine and COVID-19 highlights the need for more robust collection of data on tobacco use in medical records.

As part of its public relations strategy, the tobacco industry donated supplies, ranging from ventilators to personal protective equipment, to various countries worldwide [109]. Nevertheless, these so-called corporate social responsibility activities were conducted to improve the image of the tobacco industry and further its commercial interests. In some cases, interference from the tobacco industry influenced governmental decisions. For example, vape shops were deemed essential in Italy after the tobacco industry’s lobbying to the government [86]. In Argentina, Brazil and Russia, the tobacco industry undermined COVID-19 restrictions through media appeals and campaigns to garner public support [86]. Such interference by the tobacco industry in public health matters is alarming and highlights the need for government and regulatory bodies to remain vigilant in countering the spread of misinformation. In particular, the WHO Framework Convention on Tobacco Control recognises a ‘fundamental and irreconcilable conflict between the tobacco industry’s interests and public health policy interests’ [110]. Notably, Article 5.3 of the FCTC dictates that Parties are required to safeguard tobacco control policies from commercial and other vested interests, particularly those of the tobacco industry [95]. Parties to the WHO FCTC have a legal obligation to uphold their commitments to the international treaty [111].

## Policy Recommendations

The COVID-19 pandemic has been an extraordinary event, impacting people’s lives globally. Although effective vaccines and treatments have eased the burden of the pandemic, tobacco use will continue to cause significant illness and deaths. It is crucial to remain vigilant and use lessons learned from the pandemic to continue to support tobacco control policies [112].

The World Heart Federation recommends adopting the following measures to strengthen tobacco control in the post-COVID-19 era and in anticipation of potential future pandemics, ensuring a robust tobacco control response during health emergencies:

A. For healthcare professionals and institutions.

**Table:** 


MEASURES	OBJECTIVES

Document systematically the use of tobacco and nicotine products in medical records	To generate robust data for measuring the association and impact of tobacco on specific diseases.

Study the interplays between tobacco use, cardiovascular disease, and emerging SARS-CoV-2 variants	To inform the public, health authorities, and governments of potential additional risks. To implement timely and responsive tobacco control policies that further protect the public, including smokers, from emerging variants of interest or concern.

Ensure availability, accessibility, and affordability of tobacco cessation services, including during health emergencies	To reduce the impact of tobacco on noncommunicable diseases (e.g., cardiovascular disease), communicable diseases (e.g., COVID-19), and potential future pandemics. To support individuals seeking to quit tobacco, including during health emergencies.

Leverage digital health solutions to enhance tobacco cessation services	To improve and ensure availability, accessibility, and affordability of tobacco cessation services at all times.


B. For governments and civil society.

**Table:** 


MEASURES	OBJECTIVES

Accelerate the implementation and expansion of tobacco control policies	To capitalise on the momentum and political will to enact tobacco control regulations. To minimise the impact of tobacco on future pandemics.

Maintain tobacco control policies instituted during health emergencies	To sustain progress made to protect public health, even beyond health emergencies.

Prioritise health equity in national pandemic responses	To promote health equity for marginalised and/or vulnerable populations during health emergencies. To promote healthy lifestyles in stressful situations, such as pandemics. To address the social determinants of health in national emergency preparedness and response plans.

Implement Article 5.3 of the WHO FCTC* General Obligations: Conflicts of Interests, recognising that there is an irreconcilable conflict between the tobacco industry’s interest and public health policy interest.	To prevent tobacco industry interference in national pandemic responses, including through so-called corporate social responsibility activities. To protect tobacco control laws, including pandemic-specific policies, from vested and commercial interest of the tobacco industry.

Monitor and oversee the activities of the tobacco industry during health emergencies	To prevent the dissemination of unsubstantiated information that may lead to avoidable health complications and deaths. To prevent the tobacco industry from undermining tobacco control during national pandemic responses.

Implement Article 6 of the WHO FCTC* Price and Tax Measures to Reduce the Demand for Tobacco	To reduce affordability and thereby discourage the consumption of tobacco and nicotine products in the population. To generate revenue for government to allocate towards pandemic recovery initiatives.

Implement Article 8 of the WHO FCTC* Protection from Exposure to Tobacco Smoke	To protect the population from second-hand smoke. To prevent the potential transmission of airborne infectious agents.

Implement Article 12 of the WHO FCTC* Education, Communication, Training, and Public Awareness	To raise awareness on the interplays between tobacco, cardiovascular disease, and COVID-19 or other infectious pathogens. To educate and protect the general population from misinformation.

Implement Article 14 of the WHO FCTC* Demand Reduction Measures Concerning Tobacco Dependence and Cessation	To reduce the impact of tobacco on noncommunicable diseases (e.g., cardiovascular disease), communicable diseases (e.g., COVID-19), and potential future pandemics. To support individuals seeking to quit tobacco, including during health emergencies.


*Countries that are not signatories to the WHO Framework Convention on Tobacco Control are also able to enact the above measures.

## Conclusion

The COVID-19 pandemic has had profound direct, indirect and long-term impacts on individual and public health, as well as health systems.

A myriad of studies have demonstrated that tobacco use, a major risk factor for cardiovascular disease, increases the severity and mortality rates associated with COVID-19, as well as the probability of developing long COVID. Evidence also indicates that people living with cardiovascular disease experience worse outcomes from COVID-19, largely due to its intrinsic thrombotic complications. As such, the triad of tobacco, COVID-19, and cardiovascular disease constitutes a dangerous mix. It will also be essential to closely monitor any shifts in these interplays, as SARS-CoV-2 continues to mutate.

Lockdowns and social distancing measures have affected multiple aspects of our lifestyles and mental health, all of which have implications for cardiovascular health. In addition, the disruption of health services, stemming from fears of virus transmission and redirection of essential resources, became a hallmark of the impact of the pandemic on health systems. In particular, services related to cardiovascular disease and tobacco cessation faced significant interruptions.

Advancing tobacco control was especially challenging in the context of the pandemic. Although some countries have commendably enacted a number of COVID-specific tobacco control measures, additional actions could have further alleviated tobacco and COVID-19-related morbidity and mortality. The WHO Framework Convention on Tobacco Control provides a range of solutions to many of the challenges encountered during the pandemic, especially in the areas of tobacco cessation and tobacco industry interference. Lessons from the COVID-19 crisis can help the world better prepare for future pandemics. Considering the impact of tobacco on the COVID-19 pandemic, it is crucial for tobacco control to remain a central element of pandemic preparedness and response.
